# Is the New EN689 a Better Standard to Test Compliance With Occupational Exposure Limits in the Workplace?

**DOI:** 10.1093/annweh/wxab111

**Published:** 2021-12-02

**Authors:** Antonio D’Errico, Remko Houba, Hans Kromhout

**Affiliations:** 1 Institute for Risk Assessment Sciences (IRAS), Utrecht University, Utrecht, The Netherlands; 2 Cancer Epidemiology Unit, Department of Medical Sciences, University of Torino, Torino, Italy; 3 Netherlands Expertise Centre for Occupational Respiratory Disorders (NECORD), Utrecht, The Netherlands

**Keywords:** BOHS-NVvA, compliance, EN689, measurement strategy, occupational exposure limits, preliminary test, similar exposure group, statistical test

## Abstract

**Objective:**

To evaluate the performance of three measurement strategies to test compliance with occupational exposure limits of similarly exposed groups (SEGs): the old and new versions of EN689, and the BOHS-NVvA guidance on measuring compliance.

**Methods:**

Respirable dust exposures concentrations (*n* = 1383) measured within the member companies of IMA-Europe were used to compare compliance decisions between the three measurement strategies. A total of 210 SEGs of which 158 with repeated measurements were analysed. An R studio *OHcomplianceStrategies* package was created for the purpose.

**Results:**

The old EN689 strategy resulted in the highest number of compliant SEGs in the preliminary tests and statistical test (49–52% and 83%) with lower percentages of compliance with the new EN689 standard (32–44% and 71%). The percentage of non-compliant SEGs was relatively similar between the old and new EN689 for the preliminary tests (1–12% versus 6–11%). However, the new EN689 declared almost twofold more SEGs non-compliant when applying the statistical test (29% versus 17%). The BOHS-NVvA individual test showed results in between the 26% non-compliant SEGs.

**Conclusion:**

This study showed differences in compliance decisions between the old and new EN689, with the new EN689 being considerably more stringent and resulting in more non-compliant SEGs.

## What’s Important About This Paper?

Performance of the recently updated European method to assess compliance with occupational exposure limits for exposures to airborne substances in the workplace (EN689) was tested for 1383 full-shift respirable dust measurements collected among 867 workers employed in 210 similar exposure groups (SEGs). It showed that based on the same exposure data the new EN-689 was considerably more stringent and resulted in more non-compliant SEGs than the 1995 version of EN689. The BOHS-NVvA individual test taking into account within- and between-worker variability in exposure concentrations showed non-compliance percentages in between the two versions of EN-689.

## Introduction

The European standard EN689 was developed in 1995 (EN689, 1995) and updated in 2018 (EN689, 2018) to harmonize methods to assess compliance with occupational exposure limits (OELs) for exposures to airborne substances in workplaces. The compliance assessment of workers’ exposures is performed per SEG through the application of several standardized tests. In addition to group compliance per SEG, the BOHS-NVvA (British and Dutch Occupational Hygiene Societies) guidance of 2011 (BOHS-NVvA, 2011) outlined a method to test compliance for an individual worker’s exposure, which was demonstrated to be an important element of compliance assessment missing in the European standard (Kromhout *et al*., 1993; Ogden and Lavoué, 2012). We performed a study to observe differences in results of compliance testing between EN689-1995, EN689-2018, and the BOHS-NVvA individual test.

## Methods

Compliance strategies (see Supplementary Table 1, available at *Annals of Work Exposures and Health* online).

### EN689 preliminary test

Equations comparing exposure concentrations with the OEL are reported in the Annex C of EN689:1995 and Section 5.5.2 of EN689:2018.

### EN689 statistical test

Statistical models are described in the Annex D of EN689:1995 and Annex F of EN689:2018. The statistical principle of EN689:1995 was partially modified to compare the two standards. *The green and orange situation* were omitted and the conventional 5% probability of exceeding the OEL was used to determine compliance or non-compliance of SEGs over lognormal distributions.

### BOHS-NVvA individual compliance test

In Chapter 3 of the BOHS-NVvA guidance, analysis of variance is used to determine the variability of individuals’ exposures within a SEG and consequently test compliance of an individual worker’s exposure with the OEL. To compare the individual compliance test with the statistical tests of the EN689s, a minimum of six measurements for each SEG with at least one worker with repeated measurements was considered.

### Dataset

One thousand three hundred and eighty-three full-shift respirable dust measurements of 867 workers collected within the member companies of the European Industrial Mineral Association (IMA-Europe) (Zilaout *et al*., 2020) were used in the comparison. A total of 210 groups of workers (SEGs), of which 158 groups with repeated measurements of the same worker, were analysed. The SEGs were determined through the criteria reported in the Respirable Crystalline Silica Monitoring Protocol of NEPSI (European Network for Silica). Each group of workers presented a minimum of six measurements in accordance with EN689:1995, Annex D. The SEGs were obtained from six sampling campaigns at six sites, representing nine different jobs. The measurements analysed in the preliminary tests were progressively selected per sampling date from the SEGs.

### Statistical analysis

R studio (version 1.3.959) *OHcomplianceStrategies* package (https://github.com/tonyderrico/OHcomplianceStrategies) was built and used to apply the strategies for testing compliance detailed in both versions of EN689 and BOHS/NVvA. We assumed an OEL for respirable dust of 1 mg m^−3^.

## Results

The numbers and percentages of compliance, uncertain compliance, and non-compliance decisions for the European and BOHS-NVvA strategies are presented for all SEGs (Table 1) and for the SEGs with repeated measurements (Table 2), respectively. Uncertain compliance is only relevant for the preliminary phase for situations in cases where no final decision can be made yet, due to the limited number of samples.

**Table 1.** Compliance parameters per 210 similar exposed groups (SEGs). #1, #3, #4, #5 are the number of measurements needed within a SEG; 5% exc. is the 5% probability for exceedance of OEL exceedance based on the exposure measurement distribution; UTL_95,70_ is Upper Tolerance Limit 95% confidence limit, 70% confidence level and threshold probability parameter for OEL exceedance based on the exposure measurement distribution; I.C. is Individual Compliance achieved when more than 80% of SEG members have at least 95% of their exposures < OEL.

	Preliminary test					Statistical test	
	EN689 1995		EN689 2018			EN689 1995	EN689 2018
210 SEGs	#1 <0.1	#3 <0.25	#3 <0.1 OEL	#4 <0.15 OEL	#5 <0.2 OEL	5% exc.	UTL_95,70_
Compliance	110 (52%)	102 (49%)	68 (32%)	74 (35%)	92 (44%)	174 (83%)	150 (71%)
Uncertain compliance	97 (47%)	83 (40%)	129 (61%)	115 (55%)	94 (45%)		
Non-compliance	3 (1%)	25 (12%)	13 (6%)	22 (10%)	24 (11%)	36 (17%)	60 (29%)

**Table 2.** Compliance parameters of the subgroup including 158 similar exposed groups (SEGs) with only repeated measurements.

	Preliminary test					Statistical test		Phase 3
	EN 689 1995		EN689 2018			EN689 1995	EN689 2018	BOHS/NVvA 2011
158 SEGs (only with repeats)	#1 <0.1	#3 <0.25	#3 <0.1 OEL	#4 <0.15 OEL	#5 <0.2 OEL	5% exc.	UTL_95,70_	I.C.
Compliance	83 (53%)	75 (47%)	49 (31%)	56 (35%)	69 (44%)	129 (82%)	111 (70%)	116 (73%)
Uncertain compliance	73 (46%)	64 (41%)	100 (63%)	87 (55%)	71 (45%)			
Non-compliance	2 (1%)	19 (12%)	9 (6%)	15 (9%)	18 (11%)	29 (18%)	47(30%)	42 (27%)

The measurement results were similar for all 210 SEGs compared with the subgroup of 158 SEGs with repeated measurements.

The EN689:1995 reported the highest percentage of groups in compliance with the OEL, which ranged from 52 to 49% in the preliminary tests and 83% in the statistical test of EN689:1995. The revised EN689 showed lower percentages of compliance (32–44% and 71%, respectively). The largest difference of 20% was, as expected, seen for the first step in the preliminary test for a SEG based on only 1 (EN689:1995) compared to a SEG with 3 observations (EN689:2018). Increasing the number of observations in the preliminary phase resulted in lower percentages of uncertain compliance decisions in both standards (47–40% for EN689:1995 and, respectively, 61–45% for EN689:2018).

The statistical test of non-compliance (exceedance) resulted in almost twice as many non-compliant SEGs when using EN689:2018 compared with EN689:1995 (29% versus 17%).

Following the BOHS/NVvA guidance, we had to determine individual compliance (in those SEGs with relatively high between-worker variability (20% or more of the total variability) for 67 (42%) of the SEGs with repeated measurements). Individual compliance within a SEG showed non-compliance to be in between the two EN689 versions at 27% of the SEGs. A sensitivity analysis in which groups with only one worker with repeats [*n* = 26 (16%)] were removed led to a comparable percentage of non-compliant SEGs (26%).

## Discussion

Differences in compliance decisions between the old and new European standard, and BOHS/NVvA individual compliance test were investigated. Respirable dust measurements collected within SEGs from the IMA-Europe Dust Monitoring Programme were used as a basis for comparison of the results of the compliance testing. The old EN689 demonstrated to be less stringent in both the preliminary test and the statistical test when compared with the new standard. The new EN689 will encourage occupational hygienists to collect more measurements within a SEG (at least three) and will therefore improve the precision and accuracy of the risk assessment. Interestingly, in the old EN689, the number of compliant SEGs decreased with increasing number of samples in the preliminary test, suggesting doubtful decisions based on one sample only. Like for the preliminary test, the statistical test turned out to be more restrictive in the new EN689 with an almost twofold higher amount of non-compliant SEGs compared with the old EN689 clearly due to the introduction of UTL_95,70_ value to base a compliance decision upon. Similar percentages but slightly lower percentages of non-compliance were reported with the individual compliance strategy propagated by the BOHS-NVvA guidance.

Individual compliance testing does require repeated measurements within a SEG and explicitly takes into account temporal and individual variability in (average) exposure within a SEG (Kromhout *et al*., 1993; Rappaport *et al*., 1995). Eventually incorporating individual compliance testing within EN689 will further improve the quality of assessment of workers health risks due to exposure to chemical agents and process generated substances (Clerc *et al*., 2014).

## Conclusion

This study showed clear differences in compliance decisions between the old and new EN689. The largest differences were seen for the number of compliant SEGs in the preliminary tests and for non-compliance in the statistical test. Both clearly showed that the revised EN689 is considerably more stringent than the original EN689. It is clear that the limited number of measurements proposed in the original EN689 could easily result in more doubtful decisions during the preliminary tests. Incorporating the individual compliance test, that takes into account between-worker differences in exposure, into a next version of EN689 will result in even further improvement.

## Supplementary Material

wxab111_suppl_Supplementary_Table_S1

## Data Availability

The data underlying this article were provided by IMA-Dust Monitoring Programme. Data will be shared on request to the corresponding author only after the permission of IMA-Europe.
