# Supplementary material for: Is the New EN689 a Better Standard to Test Compliance With Occupational Exposure Limits in the Workplace?
Source: Ann Work Expo Health. 2021 Dec 2;66(3):412–5. doi: 10.1093/annweh/wxab111 (PMC8922169; doi:10.1093/annweh/wxab111)
Supplement: wxab111_suppl_Supplementary_Table_S1 [file wxab111_suppl_supplementary_table_s1.docx]

*Supplementary Material*

Is the new EN689 a better standard to test compliance with Occupational Exposure Limits in the workplace?

*Antonio D’Errico^a,b^; Remko Houba^a,c^, Hans Kromhout^a*^*

*^a^ Institute for Risk Assessment Sciences (IRAS), Utrecht University, Yalelaan 2, 3584 CM Utrecht, the Netherlands.*

*^b^ Cancer Epidemiology Unit, Department of Medical Sciences, University of Torino, Via Santena 7, 10126, Torino, Italy*

*^c^ Netherlands Expertise Centre for Occupational Respiratory Disorders (NECORD), Utrecht, the Netherlands.*

*^*^Author to whom correspondence should be addressed. e-mail: h.kromhout@uu.nl*

*Supplementary Table 1 - Functions and statistical models used to compare the EN689s and the individual test of BOHS/NVvA 2011*

|  | **Preliminary Test** | **Statistical Test** | **Individual Compliance Test** |
| --- | --- | --- | --- |
| **EN689:1995** | **C**  S_1_ ≤ 0.1*OEL;  all S_3…n_ ≤ 0.25*OEL;  all S_3…n_ ≤ 1*OEL or GM ≤ 0.5  **UC**  S_1_ ≤ 1*OEL and > 0.1*OEL;  if any of S_3…n_ > 0.25 or GM > 0.5  **NC**  S_1_ > 0.1*OEL;  if any of S_3…n_ > OEL or > 1*OEL | **C**  SEG P_r_ of exceeding the OEL ≤ 5% over a lognormal distribution  **NC**  SEG P_r_  of exceeding the OEL > 5% over a lognormal distribution |  |
| **EN689:2018** | **C**  all S_1,2,3_ ≤ 0.1*OEL;  all S_1,2,3,4_ ≤ 0.15*OEL;  all S_1,2,3,4,5_ ≤ 0.2*OEL  **UC**  if one S_1,2,3_ > 0.1*OEL and the others ≤ 0.1*OEL;  if one S_1,2,3,4_ > 0.15*OEL and the others ≤ 0.15*OEL;  if one S_1,2,3,4,5_ > 0.2*OEL and the others ≤ 0.2*OEL  **NC**  if any S_1,2,3_ > OEL;  if any S_1,2,3,4_ > OEL;  if any S_1,2,3,4,5_ > OEL | **C**  SEGs P_r_ of exceeding the OEL ≤ 5% with at least 70% confidence level over a lognormal distribution  **NC**  SEGs P_r_  of exceeding the OEL > 5% with at least 70% confidence level over a lognormal distribution |  |
| **BOHS/NVvA 2011** |  |  | **C**  { [ log (OEL) – (log GM_SEG_ + 1.645 ww) ] / bw }  < 0.2  **NC**  { [ log (OEL) – (log GM_SEG_ + 1.645 ww) ] / bw }  > 0.2 |

**Table 1.** Functions and statistical models used to compare the EN689s and the individual test of BOHS/NVvA 2011. S = Sample, C = Compliance, UC = Uncertain Compliance; NC = Non-Compliance; GM = Geometric Mean; OEL = Occupational Exposure Limit; SEG = Similar Exposed Group; ww = standard deviation within workers; bw = standard deviation between workers.
